# Seasonal modulation of antibody response to diphtheria-tetanus-pertussis vaccination in infants: a cohort study in rural Gambia

**DOI:** 10.1186/s12889-021-11383-7

**Published:** 2021-07-22

**Authors:** Sandra G. Okala, Momodou K. Darboe, Fatou Sosseh, Bakary Sonko, Tisbeh Faye-Joof, Andrew M. Prentice, Sophie E. Moore

**Affiliations:** 1 Department of Women and Children’s Health, King’s College London, St Thomas’ Hospital, 10th Floor North Wing, London, SE1 7EH UK; 2 MRC Unit The Gambia at the London School of Hygiene and Tropical Medicine, Banjul, The Gambia

## Abstract

**Background:**

In rural Gambia, rates of malnutrition and infection are higher during the annual rainy/‘hungry’ season (June–October) in comparison to the dry/‘harvest’ season (November–May). The effects of this seasonal pattern on an infant’s immune development and their capacity to respond to childhood vaccinations remain unclear. The aim of the current analysis was to determine whether antibody responses to diphtheria-tetanus-pertussis (DTP) vaccinations in infants differ between seasons.

**Methods:**

Infants received the DTP vaccine at 8, 12 and 16 weeks of age and antibody titres were measured in blood samples collected at 12 (*n* = 710) and 24 (*n* = 662) weeks of age. Mean DTP antibody titres, adjusted for maternal and infant confounders, were compared by *t*-tests and the effect sizes of the mean differences were calculated between seasons at mid-gestation (20 weeks gestation) and first vaccination (8 weeks of infant age).

**Results:**

A smaller number of infants received their first vaccination during the rainy/hungry season months compared to the dry/harvest season (*n* = 224 vs. *n* = 486). At 12 weeks, infants vaccinated during the rainy/hungry season had lower weight-for-length Z-scores (*p* = 0.01) and were more likely to be anaemic (*p* < 0.001). Their mothers, however, were pregnant mostly during the dry/harvest season, had higher weight gain (*p* < 0.001) and were less likely to be anaemic during pregnancy (*p* < 0.001). At 12 weeks, infants vaccinated during the rainy/hungry season had significantly higher mean diphtheria, tetanus and pertussis antibody titres; by 62.3, 16.9 and 19.7%, respectively (all, *p* < 0.001). However, at 24 weeks, they had lower mean anti-diphtheria titres (by 20.6%, *p* < 0.001) compared with infants vaccinated during the dry/harvest season, and no differences were observed in mean tetanus and pertussis antibody titres by vaccination season.

**Conclusions:**

Infant antibody response to the primary dose of the DTP vaccine was influenced by both season of pregnancy and infancy, although effects were diminished following three doses. Environmental exposures, including nutrition, to both the mother and infant are hypothesised as likely drivers of these seasonal effects.

**Supplementary Information:**

The online version contains supplementary material available at 10.1186/s12889-021-11383-7.

## Background

Seasonal variation in agricultural food production is a major concern for subsistence farming populations across the world impacting on their income, nutritional intake and health outcomes [11]. In sub-Saharan Africa, two-thirds of the population, representing 645 million people in 2018, live in rural areas and rely predominantly on subsistence farming for their livelihood (World Bank). In this setting, rainfed agriculture accounts for 95% of food production and low harvests, alongside poor long-term storage for perishable food products, put many subsistence farming households at risk from a seasonal cycle of poverty and hunger [9]. In rural Gambia, two distinct seasons; a dry season (November–May) and a rainy season (June–October) dictate many aspects of health and behaviour. The dry season is characterised by elevated temperatures, low rainfall and few infections and is referred to as the ‘harvest season’ [12]. The rainy - or ‘hungry’ - season is characterised by heavy rainfalls, an increase in agricultural workload and higher rates of infection and coincides with the end of food supplies from the previous harvest. Women are especially vulnerable to this seasonal pattern as they contribute to the agricultural labour force and continue to endure intensive physical activity during pregnancy and lactation [9]. This results in a negative energy balance [28] and leads to a higher prevalence of low birth weight infants, iron deficiency anaemia and maternal and child morbidity and mortality, primarily from infections, during the rainy season months [3, 29].

Child mortality is 15 times higher in sub-Saharan Africa compared to high-income settings with infectious diseases as one of the leading causes of death in children under-5 years of age (WHO−World Health Organization). Although vaccinations have been effective in reducing child mortality [21], undernutrition, which has been identified as the underlying cause of nearly half of infectious-disease related deaths in children [2], continues to hamper vaccination efforts. This is especially relevant in early infancy, the age when over half of child deaths occur as the immune system is still developing and most vulnerable [15]. Despite close links between seasonality and nutritional and health outcomes, few studies have examined the impact of season on the ability to respond to childhood vaccinations. Earlier research has yielded mixed findings, both with respect to the existence of any seasonal effects and, in studies where an effect was observed, in relation to the season of peaks in vaccine response [26].

The present study aimed to determine whether antibody responses to diphtheria-tetanus-pertussis (DTP) vaccinations in infants varied by season in a mother-infant cohort from rural Gambia. Season of mid-gestation was selected a priori as the exposure variable.

## Methods

### Study design and participants

The current study is a secondary analysis of data collected during a randomized clinical trial of nutritional supplementation during pregnancy and infancy conducted in the West Kiang region of The Gambia; the Early Nutrition and Immune Development (ENID) Trial (ISRCTN49285450). The trial protocol has been published and described in detail elsewhere [23]. Infant antibody response to vaccination was measured as a secondary outcome in the trial, and the impact of maternal nutritional supplementation during pregnancy on infants’ antibody responses has been presented previously [24]. Key details pertaining to the current analysis are included here; full details can be found elsewhere [23, 24]. Briefly, all women of reproductive age (18 to 45 years) registered in the West Kiang Demographic Surveillance System (DSS) [12] were invited to participate in the trial, and eligible women enrolled (Figure S1). Exclusion criteria from the trial included pregnancy over 20 weeks gestation, multiple pregnancy, severe anaemia (haemoglobin (Hb) < 7 g/dL) and confirmed HIV positive.

### Interventions and assessments in pregnancy

Enrolled pregnant women were randomised to one of four nutrition intervention groups (as detailed in Table S1), with daily supplementation from enrolment to delivery. During pregnancy, women were seen at the Medical Research Council (MRC) Unit The Gambia Keneba field station at enrolment, and 20 and 30 weeks gestation for a standard pregnancy examination. Maternal anthropometry and ultrasound measures of foetal biometry were taken by a study midwife at each visit. Foetal size at the enrolment visit was used to estimate gestational age. All measurements were performed using standard operating procedures and standardised validated equipment.

### Interventions and assessments in infancy

Study midwives visited all women after delivery for a standard health examination and anthropometric measurements of the newborn (weight, length, mid-upper arm circumference [MUAC], and head circumference). Infants were then seen at 1, 8, 12, 24, and 52 weeks of age at the MRC Keneba field station and at 16, 20, 32, and 40 weeks of age at home for sample collections, anthropometric measurements and health assessments. The same standard and regularly validated anthropometric equipment was used at each visit (clinic and home visits). At these infant visits, EPI vaccines were also administered according to the Gambian Government guidelines [23], full details of which are given elsewhere [24]. Here, we present antibody response to the diphtheria-tetanus-pertussis (DTP) vaccines. As part of the EPI programme, these vaccines are given at 8, 12 and 16 weeks of infant age; here, antibody responses were measured in blood samples collected at 12 and 24 weeks of age reflecting infant responses to the first dose of vaccine given at 8 weeks (in samples measured at 12 weeks) and infant antibody responses after all three doses (at 8, 12, and 16 weeks) of the vaccine (in samples measured at 24 weeks). Data on maternal morbidity (during pregnancy only) and infant morbidity and feeding practices (from birth to 52 weeks) were obtained from questionnaires administered weekly.

From 6 months of age, infants were further randomised to receive either an unfortified LNS paste or the same formulation fortified with MMN. For the present analysis, however, the infant intervention arms will not be examined, as the outcomes included were assessed before infant supplementation.

### Laboratory analyses

All antibody assays were performed at the MRC Unit The Gambia. Serum-specific IgG antibody responses directed against the three components of the DTP vaccine: diphtheria toxoid (Dtxd), tetanus toxin (Ttx) and pertussis toxin (Ptx) were measured with a validated multiple immunoassay based on Luminex xMAP technology [17, 18]. Full details of the laboratory procedures are published elsewhere [24].

### Covariates

Information collected in the West Kiang DSS was used to verify maternal age and date of birth. A questionnaire collected at the first visit was used to generate maternal parity, defined as the number of previous pregnancies (live births and stillbirths), and a binary variable for formal education (Yes/No), based on whether women attended at least a year in an Arabic/and or English school. Maternal anaemia was defined as Hb level < 11 g/dL according to the WHO definition. Maternal body mass index (BMI) was computed as weight (kg)/height (m)^2^. Gestational weight gain was expressed in kg/week and generated by subtracting maternal weight between three time periods; enrolment and 20 weeks gestation, 20 and 30 weeks gestation, and enrolment and 30 weeks gestation, and by dividing each subtraction by the number of weeks between measurements. Maternal morbidity was defined as the total number of morbidity episodes during pregnancy divided by the number of weeks enrolled in the trial. The amount of supplement remaining in the jars (empty, half-empty, and full) for LNS products (PE and PE + MMN) and the count of tablets left in the bottle for tablets (MMN and FeFol) were used to generate a supplementation compliance score for each woman. Maternal compliance was expressed as a percentage and calculated by dividing the total number of LNS jars or tablets consumed, by the number received and multiplying by 100.

Low birth weight (LBW) was defined as birth weight < 2500 g according to WHO guidelines. Small-for-gestational-age (SGA) was defined as a birth weight-for-gestational-age below the 10th percentile of the INTERGROWTH-21ST standards for birth weight [36]. Weight-for-length-z-scores (WLZ) at 8, 12 and 24 weeks were generated using the WHO Child Growth Standards [38]. A score for infant morbidity was computed as the total number of days of reported sickness in a weekly questionnaire completed by the infant’s caregiver. At the same weekly visit, information on infant feeding practices was also collected by questionnaire. Using these data, a binary variable was generated for exclusive breastfeeding (EBF) (yes/no) to 12 (or 24) weeks of infant age.

Separate binary variables were generated to represent the season of mid-gestation and season of vaccination and defined as rainy/hungry = June to October and dry/harvest = November to May, based on the season at the 20 weeks gestation visit and the season of the first DTP vaccination at 8 weeks of infant age, respectively. To examine the impact of seasonality as a continuous variable, data were fitted using the first two pairs of the Fourier terms: denoted as F1 = sin(θ) and cos(θ) and F2 = sin(2θ) and cos(2θ), with θ representing the angle in radians of the date in relation to its position on the annual cycle (on 1 January, θ = 2π/365; on 31 December, θ = 2π).

### Statistical analysis

**Fig. 1:**
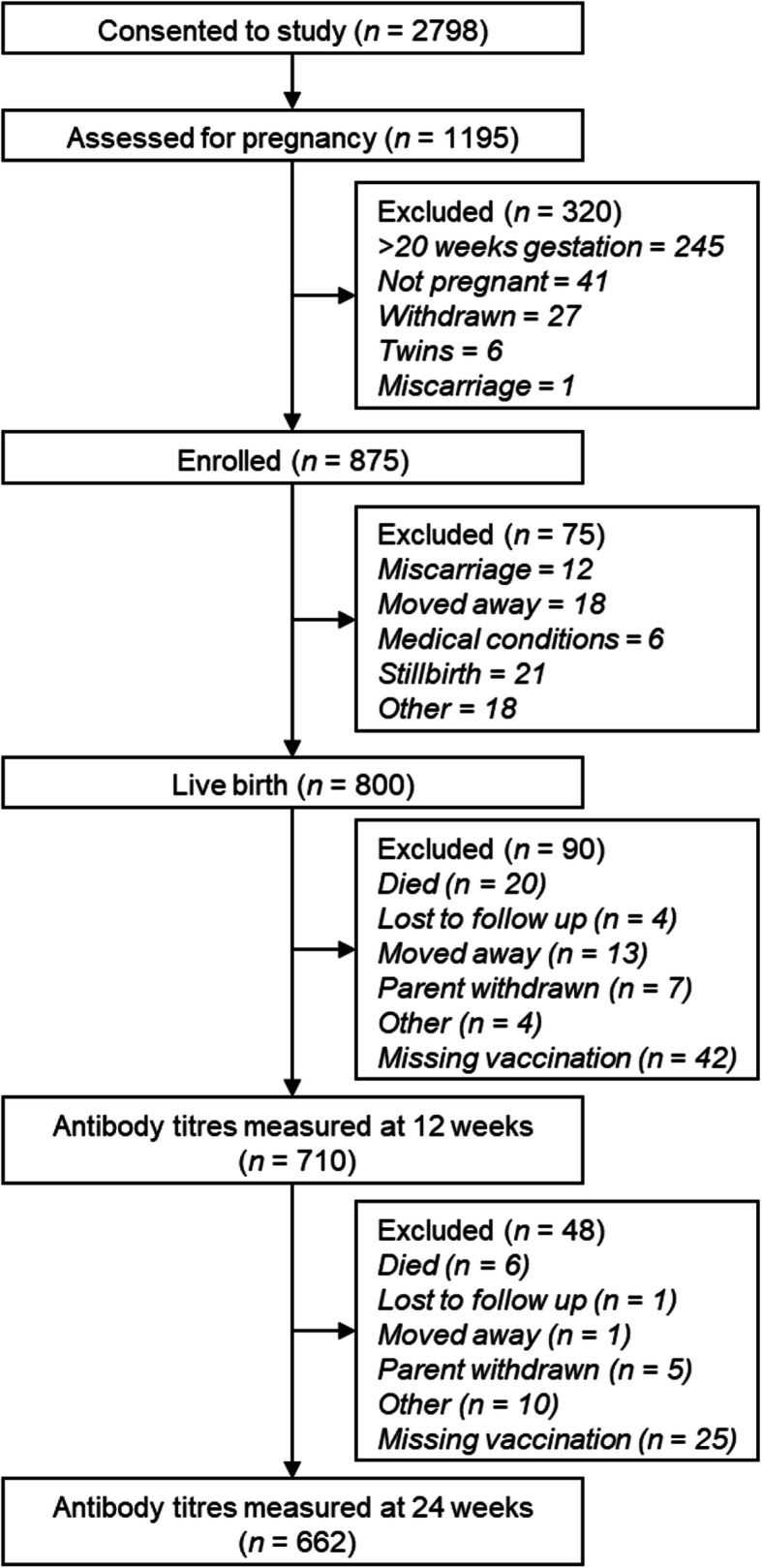
Flow diagram of participants included in the analyses

The original ENID trial was powered based on the thymic index as the primary outcomes. The current paper represents a secondary analysis by intention-to-treat of the ENID data including all mother-infant pairs with available antibody data at 12 weeks (*n* = 710) and 24 weeks (*n* = 662) (Fig. 1). Participants’ characteristics were compared by season of mid-gestation and season of first DTP vaccination using chi-squared tests for categorical variables or ANOVA for continuous variables.

Plasma antibody concentrations were skewed as assessed by the Shapiro–Wilk test for normality; therefore, the logarithms (base 10) values of the antibody titres were used for analysis. Means of antibody titres were calculated using linear regression models with robust standard errors and with or without adjustment with confounding factors. Unadjusted data analysis can be found in Table S2. The use of robust analysis minimised potential deviations from the assumptions of the multiple linear regression models, however, further verifications were performed to the models for linearity and multicollinearity and the residuals were examined to confirm homoscedasticity and normality. Log-transformed mean antibody titres by season of mid-gestation (20 weeks) or season of infant first DTP vaccination were compared by Student *t*-test and back-transformed from the log scale. Findings from previous studies and biological plausibility were used to select confounding factors [7, 17]. Maternal variables included in the linear models were as follows: age and BMI (at baseline), maternal education, supplement group, compliance to supplement, Hb levels, and maternal morbidity. Infant variables included in the models were gestational age at delivery; Fourier terms for month of mid-gestation or vaccination, sex, infant size (WLZ), and Hb levels, EBF and infant morbidity. Effect sizes of the mean difference in DTP antibody titres by season of mid-gestation or first vaccination were determined using the residual mean difference between the dry/harvest season and rainy/hungry season from the Student *t*-test and expressed as percentages (%). In additional analysis, mean DTP antibody titres were compared by both season of first vaccination and maternal supplement groups (Figure S2). The monthly variation in DTP antibody responses by the month of mid-gestation and month of first vaccination were examined by fitting the first two pairs of Fourier terms as described. All statistical analyses were performed using STATA 15·0 (StataCorp LP TX).

### Ethics

The Joint Gambian Government/MRC Unit The Gambia ethics committee approved the ENID trial (SCC1126v2). All participants provided written informed consent and the trial respected the current version of the Helsinki Declaration and Good Clinical Practice Standards.

## Results

Of the 800 live births in the ENID Trial, 710 (88.8%) infants had DTP antibody measurements available at 12 weeks of age. As previously reported, no differences were observed in participant characteristics between those remaining in the study and those lost to follow up (see [24] Supplementary Tables 1 and 2 for full details). Table 1 describes maternal and infant characteristics split by dry/harvest and rainy/hungry seasons of first DTP vaccination. The majority of infants (*n* = 486, 68.5%) were vaccinated during the dry/harvest season. Infants vaccinated during the rainy/hungry season were more likely to have been exposed to the dry season during foetal development compared with infants vaccinated during the dry season. All mothers of infants vaccinated during the rainy/hungry season were at mid-gestation (20 weeks gestation) in the dry/harvest season compared with less than a quarter of mothers of infants vaccinated during the dry/harvest season (100% vs 22.8%, *p* < 0.001). Mothers of infants vaccinated during the rainy/hungry season had higher haemoglobin levels and were less likely to be anaemic at enrolment, 20 or 30 weeks gestation compared with mothers of infants vaccinated during the dry season (all, *p* < 0.05). They were also less likely to be underweight at 20 weeks gestation (9.5% vs 4.1%, *p* = 0.01), had a higher BMI at 30 weeks gestation (23.4 ± 3.4 vs 22.9 ± 3.2 kg/m^2^, *p* = 0.045) and a higher weight gain per week across pregnancy (0.35 ± 0.15 vs 0.31 ± 0.18 kg/wk., *p* < 0.001). However, at 12 weeks of age, and compared with infants vaccinated during the dry/harvest season, infants vaccinated during the rainy/hungry season had lower haemoglobin levels (10.3 ± 1.3 vs 10.9 ± 1.4, *p* = 0.001), were more likely to be anaemic (21.2% vs 7.3%, *p* = 0.01), and were also more likely to be undernourished as observed by lower WLZ scores (− 0.51 ± 1.2 vs − 0.27 ± 1.1, p = 0.01).. Participants were evenly distributed between nutritional supplement groups.

**Table 1:** Participant characteristics by season of vaccination

	Season of vaccination (***N*** = 710)^**a**^		
	Dry/Harvest season (Nov − May) (***n*** = 486)	Rainy/Hungry season (Jun − Oct) (***n*** = 224)	***p-value***
**Maternal variables**			
**Enrolment visit**			
Visit season, *n* (%): Dry	198 (40.7)	171 (76.3)	**< 0.001**
Gestational age (weeks)	13.8 ± 3.2	13.8 ± 3.7	0.971
Age (years)	30.1 ± 6.8	30.5 ± 6.7	0.433
Parity (*n*)	4.1 ± 2.7	4.3 ± 2.7	0.358
Formal education, *n* (%)	128 (26.3)	33 (14.7)	**0.001**
Hb (g/dL)	11.3 ± 1.4	11.6 ± 1.4	**0.012**
Anaemia, *n* (%)^*b*^	190 (39.1)	68 (30.4)	**0.024**
BMI (kg/m^2^)	21.1 ± 3.4	21.2 ± 3.7	0.674
Underweight (BMI < 18.5), *n* (%)	57 (11.8)	27 (12.2)	0.898
Supplement groups, *n* (%)			
FeFol (control)	25.7 (125)	23.7 (53)	0.949
MMN	25.1 (122)	25.5 (57)	
PE	23.7 (115)	24.6 (55)	
PE + MMN	25.5 (124)	26.4 (59)	
**20 weeks gestation visit**			
Visit season, *n* (%): Dry/harvest	111 (22.8)	224 (100.0)	**< 0.001**
Hb (g/dL)	10.8 ± 1.1	11.2 ± 1.3	**< 0.001**
Anaemia, *n* (%)^*b*^	260 (55.9)	83 (42.1)	**0.001**
BMI (kg/m^2^)	22.0 ± 3.3	22.3 ± 3.4	0.254
Underweight (BMI < 18.5), *n* (%)	46 (9.5)	9 (4.1)	**0.014**
Weight gain (enrolment─20 weeks) (kg/wk)	0.35 ± 0.35	0.42 ± 0.34	**0.009**
**30 weeks gestation visit**			
Visit season, *n* (%): Dry/harvest	199 (41.0)	179 (79.9)	**< 0.001**
Hb (g/dL)	10.6 ± 1.3	10.9 ± 1.3	**0.002**
Anaemia, *n* (%)^*b*^	264 (59.1)	107 (50.7)	**0.044**
BMI (kg/m^2^)	23.0 ± 3.2	23.5 ± 3.4	**0.048**
Underweight (BMI < 18.5), *n* (%)	11 (2.3)	1 (0.45)	0.080
Weight gain (20─30 weeks) (kg/wk)	0.30 ± 0.17	0.37 ± 0.16	**0.001**
**Across pregnancy**			
Weight gain (enrolment─30 weeks) (kg/wk)	0.31 ± 0.18	0.35 ± 0.15	**< 0.001**
GA at delivery (weeks)	40.2 ± 1.4	40.2 ± 1.5	0.639
Compliance to supplementation (%)^*c*^	87.4 ± 14.6	87.4 ± 12.9	0.981
Morbidity episodes (*n*)^d^	5.6 ± 7.2	4.5 ± 5.1	0.057
**Infant variables**			
**Birth visit**			
Birth season, *n* (%): Dry	359 (73.9)	84 (37.5)	**< 0.001**
Sex, *n* (%): male	255 (52.5)	111 (49.6)	0.470
Birth weight (kg)	2.99 ± 0.41	3.05 ± 0.38	0.137
Birth length (cm)	49.54 ± 1.96	49.79 ± 1.90	0.140
LBW^e^	41 (9.7)	12 (6.9)	0.276
SGA, *n* (%)^*f*^	159 (37.8)	51 (29.5)	0.055
**8 weeks visit**			
WLZ score	−0.26 ± 1.2	−0.38 ± 1.1	0.154
**12 weeks visit**			
Hb (g/dL)	10.9 ± 1.4	10.3 ± 1.3	**< 0.001**
Anaemia, *n* (%)^*b*^	31 (7.5)	40 (21.2)	**< 0.001**
WLZ score	−0.27 ± 1.1	− 0.51 ± 1.2	**0.011**
Morbidity episodes (*n*)^*h*^	9.9 ± 12.4	8.9 ± 11.6	0.292
Exclusively breastfed, *n* (%)	456 (93.8)	207 (92.4)	0.481
**24 weeks visit**			
Hb (g/dL)	10.5 ± 1.4	10.5 ± 1.1	0.631
Anaemia, *n* (%)^*b*^	53 (13.4)	25 (14.8)	0.657
WLZ score	−0.47 ± 1.2	−0.52 ± 1.1	0.580
Morbidity episodes (*n*)^*h*^	23.42 ± 24.5	21.76 ± 21.59	0.384
Exclusively breastfed, *n* (%)	257 (52.9)	112 (50.0)	0.475

*Abbreviations*: *BMI* Body Mass Index, *DTP* Diphtheria-tetanus-pertussis, *GA* Gestational age, *Hb* Haemoglobin, *LBW* Low birth weight, *SGA* Small-for-gestational-age

^*a*^Values are means (SD) unless stated otherwise

^b^Anaemia was defined as a Hb level between 7.0 and 10.9 g/dL (WHO)

^c^Compliance to supplementation percentage was generated by dividing the number of LNS jars or tablets the women consumed by the number she received and multiplying by 100

^d^Number of morbidity episodes between enrolment and delivery

^e^LBW (low birth weight) was defined using the WHO definition of a birth weight < 2500 g

^f^SGA (small-for-gestational-age) was defined as a birth weight-for-gestational-age below the 10th percentile INTERGROWTH-21ST for birth weight

^g^Preterm birth was defined using the WHO definition of birth before 37 weeks of completed gestation

^h^Number of days of reported sickness between birth and 12 weeks or 24 weeks

**Fig. 2:**
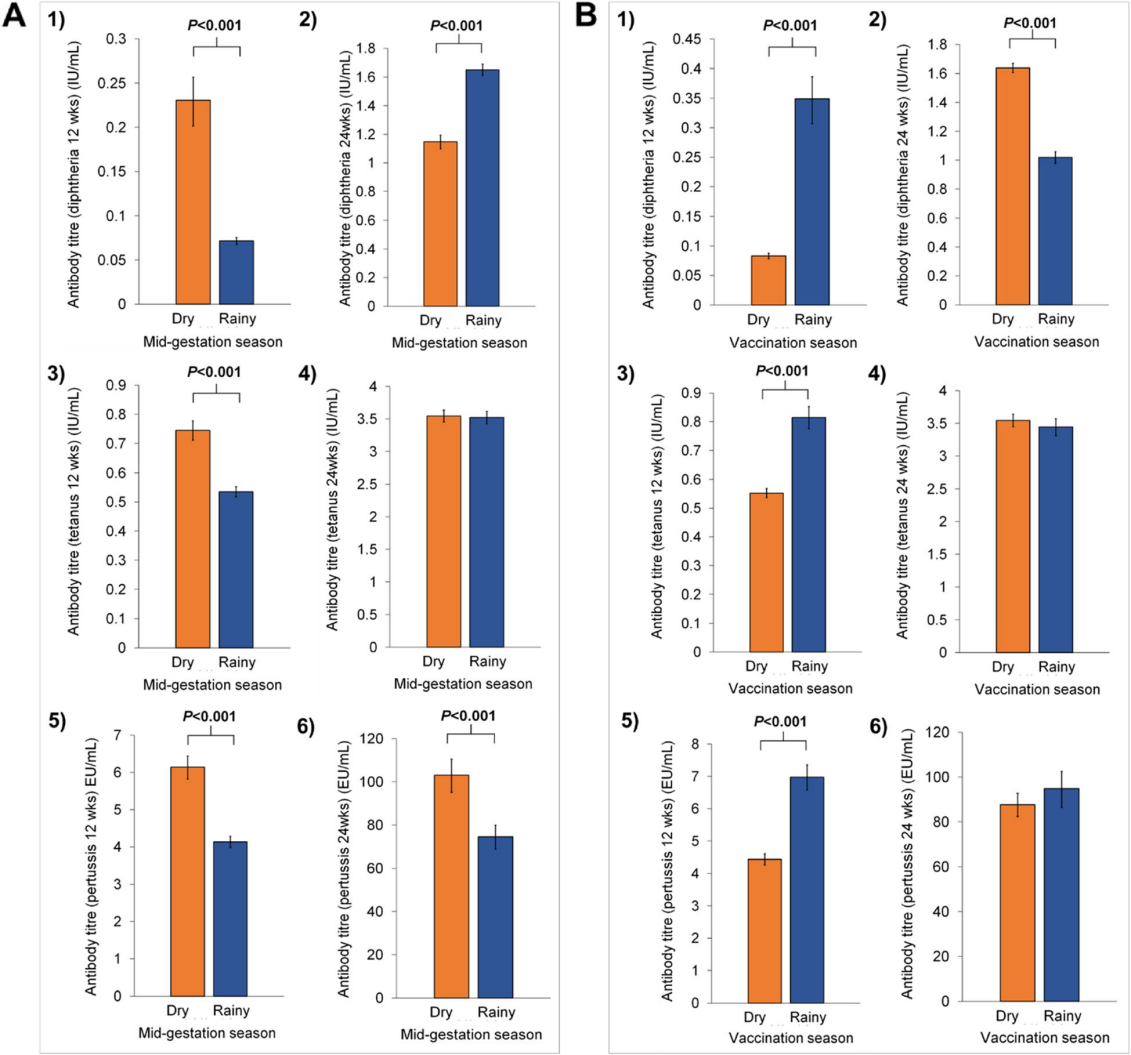
Mean (95% confidence intervals) diphtheria, tetanus and pertussis antibody titres by season of infant first DTP vaccination. The bar charts compare the means diphtheria, tetanus and pertussis antibody titres at 12 weeks (A, B and C, respectively) and 24 weeks (D, E and F, respectively) by season of first DTP vaccination in infants. In The Gambia, the dry/harvest season lasts from November to May and the rainy/hungry season from June to October. Mean antibody titres measured at 12 weeks, following the first DTP vaccination, were adjusted with maternal variables: age, BMI and formal education (yes/no) at enrolment, Hb levels at 30 weeks gestation, weight gain from enrolment to 30 weeks gestation, morbidity, supplement group and compliance to supplementation from enrolment to delivery; and with infant variables: GA at delivery, sex, WLZ at first vaccination, Hb levels at 12 weeks, morbidity, EBF (yes/no), and Fourier terms of month of mid-gestation or first vaccination. Mean antibody titres measured at 24 weeks, following the third DTP vaccination, were adjusted with the same factors mentioned above, with changes for infant WLZ at third vaccination, Hb levels at 24 weeks and morbidity and EBF (yes/no) from birth to 24 weeks

Figure 2 provides a visual comparison of the mean antibody titres by seasons and Table 2 presents the effect sizes of the mean differences in antibody titres by seasons. At 12 weeks of age, after one dose of the DTP vaccine, mean diphtheria, tetanus and pertussis antibody titres of infants exposed to the dry/harvest season at mid-gestation were all significantly higher compared to those of infants exposed to the rainy/hungry season in mid-gestation, by 50.8, 14.4 and 17.2%, respectively (all, *p* < 0.001) (Fig. 2A [panels 1, 3 and 5] and Table 2). At 24 weeks of age, after three doses of the DTP vaccine, mean diphtheria antibody titres were lower (by 15.8%, *p* < 0.001), mean tetanus antibody titres did not differ by season of mid-gestation and mean pertussis antibody titres remained significantly higher (by 14.1%, p < 0.001) in infants exposed to the dry/harvest season at mid-gestation (Fig. 2A [panels 2, 4 and 6] and Table 2). When comparing by season of infant vaccination, opposite effects were observed with lower mean diphtheria, tetanus and pertussis antibody titres at 12 weeks, in infants vaccinated during the dry season (by − 62.3%, − 16.9, − 19.7%) compared to those of infants vaccinated during the rainy/hungry season (all, *p* < 0.001) (Fig. 2B [panels 1, 3 and 5] and Table 2). At 24 weeks of age, mean diphtheria antibody titres were higher in infants vaccinated during the dry/harvest season (by 20.6%, p < 0.001). However, neither tetanus and pertussis mean antibody titres differed by season of vaccination when measured at 24 weeks of age (Fig. 2B [panels 2, 4 and 6] and Table 2).

**Table 2:** Mean diphtheria, tetanus and pertussis antibody titres in infants at 12 and 24 weeks of age by season of mid-gestation and first DTP vaccination

	Season of mid-gestation				Season of vaccination			
Vaccine	Dry/Harvest season (***n*** = 335)	Rainy/Hungry season (***n*** = 375)	Effect size (95%CI)^**a**^	***p-value***^***b***^	Dry/Harvest season (n = 486)	Rainy/Hungry season (n = 224)	Effect size (95%CI)^**a**^	***p-value***^***b***^
**12 weeks**								
Diphtheria	0.23 (0.20, 0.26)	0.07 (0.07, 0.08)	50.8 (45.2, 56.4)	**< 0.001**	0.08 (0.08, 0.09)	0.35 (0.31, 0.39)	−62.3 (−67.2, −57.4)	**< 0.001**
Tetanus	0.75 (0.71, 0.78)	0.54 (0.52, 0.55)	14.4 (12.0, 16.7)	**< 0.001**	0.55 (0.54, 0.57)	0.81 (0.78, 0.85)	− 16.9 (− 19.2, − 14.6)	**< 0.001**
Pertussis	6.1 (5.8, 6.5)	4.1 (4.0, 4.3)	17.2 (14.5, 19.9)	**< 0.001**	4.4 (4.3, 4.6)	7.0 (6.6, 7.4)	−19.7 (−22.6, − 16.7)	**< 0.001**
**24 weeks**								
Diphtheria	1.1 (1.1, 1.2)	1.7 (1.6, 1.7)	−15.8 (− 17.8, − 13.8)	**< 0.001**	1.6 (1.6, 1.7)	1.0 (0.98, 1.1)	20.6 (18.9, 22.3)	**< 0.001**
Tetanus	3.5 (3.5, 3.6)	3.5 (3.4, 3.6)	0.30 (−1.3, 1.9)	0.734	3.5 (3.5, 3.6)	3.4 (3.3, 3.6)	1.3 (−0.80, 3.3)	0.230
Pertussis	103.1 (95.7, 111.1)	74.5 (69.1, 80.3)	14.1 (9.5, 18.7)	**< 0.001**	87.7 (82.7, 93)	94.8 (87.1, 103.3)	−3.4 (−7.9, 1.1)	0.142

Note: Mean antibody titres measured at 12 weeks, following the first DTP vaccination, were adjusted with maternal variables: age, BMI and formal education (yes/no) at enrolment, Hb levels at 30 weeks gestation, weight gain from enrolment to 30 weeks gestation, morbidity, supplement group and compliance to supplementation from enrolment to delivery; and with infant variables: GA at delivery, sex, WLZ at first vaccination, Hb levels at 12 weeks, morbidity, EBF (yes/no), and Fourier terms of month of mid-gestation or first vaccination. Mean antibody titres measured at 24 weeks, following the third DTP vaccination, were adjusted with the same factors mentioned above, with changes for infant WLZ at third vaccination, Hb levels at 24 weeks and morbidity and EBF (yes/no) from birth to 24 weeks. Abbreviations: BMI, body mass index; CI, confidence interval; DTP, diphtheria-tetanus-pertussis; EBF, exclusively breastfed; GA, gestational age; Hb, haemoglobin; WLZ, weight-for-length-z-score

^*a*^Effect sizes were determined using the mean difference between the dry/harvest season and rainy/hungry season of vaccination from the Student’s *t-*test and were expressed as percentages (%)

^*b*^The p-values were calculated by Student’s *t*-test on the log-transformed antibody concentrations

In a further analysis, we compared the percentage of infants with protective vaccine responses. When assessed at 12 weeks of age, significantly more infants exposed to the dry/harvest season at mid-gestation had protective diphtheria, tetanus and pertussis antibody titres (63.0, 98.8 and 61.8%, respectively) compared with infants exposed to the rainy season (48.8, 96.0 and 53.1%; all, *p* < 0.05) (Table S3). At 24 weeks of age, most infants presented protective antibody titres, however, there were significantly fewer infants exposed to the dry/harvest season at mid-gestation with protective diphtheria vaccine response compared with infants exposed to the rainy/hungry season at mid-gestation (93.8% vs 99.7%, *p* < 0.001). At both 12 and 24 weeks of age, opposite results were observed when compared by season of vaccination.

As we previously observed differences in DTP antibody titres by maternal supplement groups [24], we further compared DTP antibody titres by both season of vaccination and maternal nutritional supplement groups (Figure S2). Differences in DTP antibody titres by season of first vaccination were generally of greater effect sizes than differences in DTP antibody titres by maternal supplement groups. However, maternal nutritional supplementation with MMN or PE + MMN was largely found to improve DTP vaccine responses and this particularly in infants vaccinated during the dry/harvest season (statistical comparisons presented in detail in Figure S2).

**Fig. 3:**
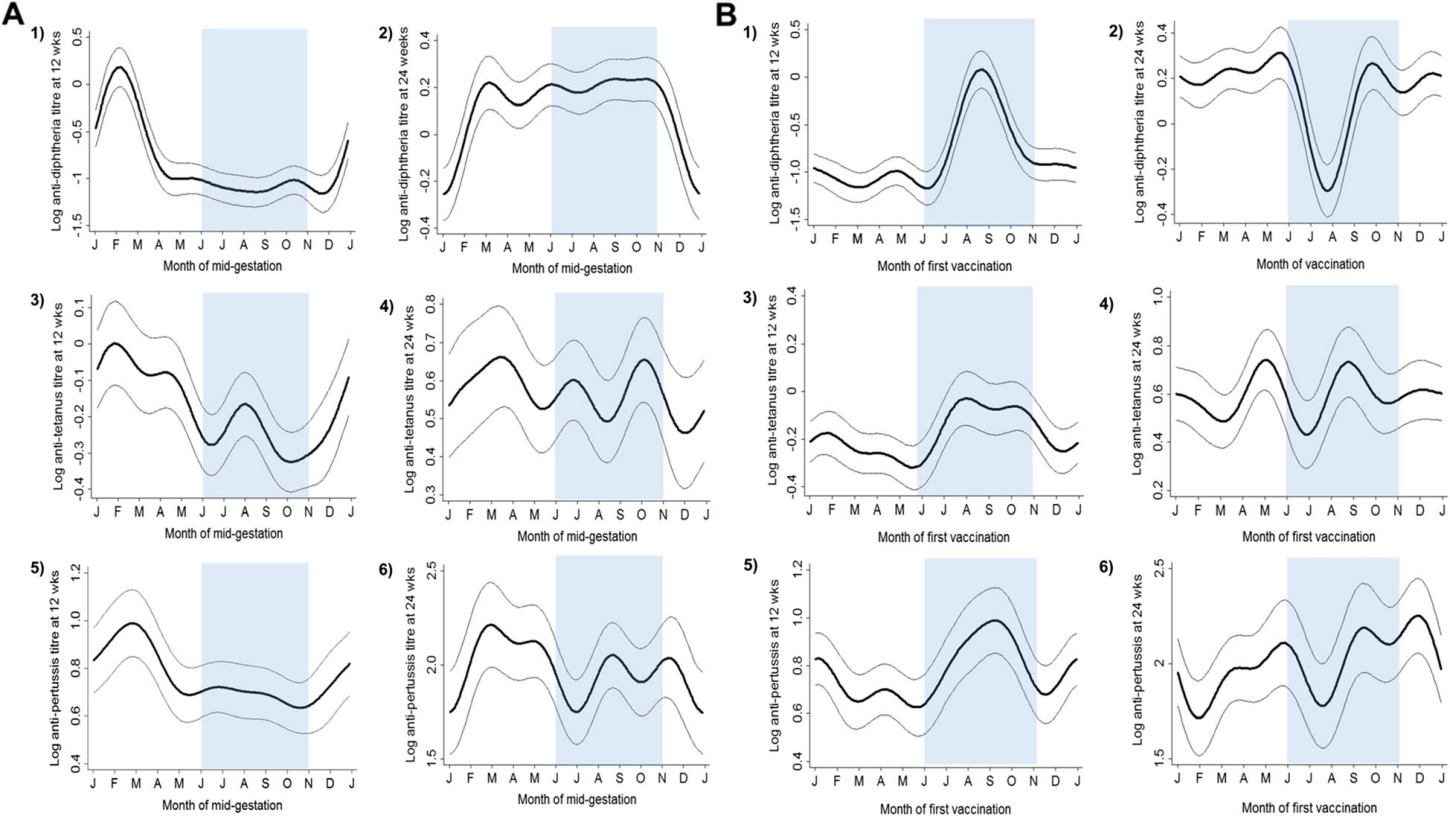
Monthly variation of log 10-transformed diphtheria, tetanus and pertussis antibody titres in infants measured at 12 (A1,3,5; B1,3,5) and 24 (A2,4,6; B2,4,6) weeks of age. The blue shading highlights the rainy/hungry season in The Gambia which lasts from June to October. The dry season runs from November to May (no shading)

We then explored how these bimodal seasonal differences translated into variations at the monthly level and examined variations in mean DTP antibody titres in infants at 12 and 24 weeks of age by both month of mid-gestation (Fig. 3A) and month of vaccination (Fig. 3B). At 12 weeks, the peaks in DTP antibody titres for the month of mid-gestation were generally observed in February (Fig. 3A, panels 1, 3 and 5), and for the month of vaccination, in September (Fig. 3B, panels 1, 3 and 5). At 24 weeks, the lowest responses were mostly observed in January for the month of mid-gestation (Fig. 3B, panels 2, 4 and 6), and in July/August for the month of vaccination (Fig. 3B, panels 2, 4 and 6).

To help understand the continuum of seasonal exposures through pregnancy and early infancy, infants exposure to the rainy/hungry season months was examined retrospectively from the month of first vaccination (at 8 weeks of infant age) to the month of conception (Table 3). As observed in Fig. 3, infants with the highest DTP antibody titres at 12 weeks were vaccinated in September which corresponds to February at mid-gestation and October at conception (Table 3). These infants had the maximal exposure to the rainy/hungry season from birth to vaccination (2 months) and the minimal exposure during foetal development (2 months), and this in the first and last month of pregnancy (Table 3).

**Table 3:**
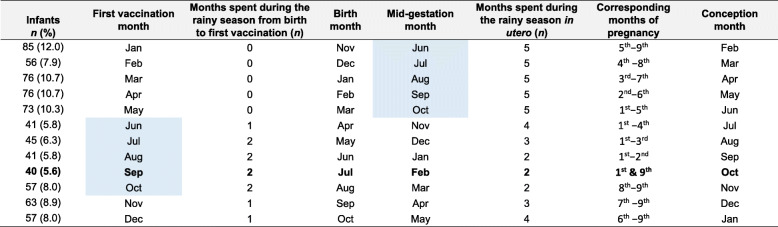
Exposure to rainy season months retrospectively from the first DTP vaccination to conception

Note: The blue shading highlights the rainy/hungry season in The Gambia which lasts from June to October. The dry/harvest season runs from November to May (no shading). The peaks in DTP vaccine antibody response were generally observed in September (highlighted in bold). Infants vaccinated during the rainy season (Jun − Oct) were more exposed to the rainy/hungry season from birth to vaccination (for 1–2 months) compared with infants vaccinated during the dry season (for 0–1 month). However, during foetal development, these infants were less exposed to the rainy/hungry season (2–4 months), and this either at the beginning and/or the end of pregnancy, compared with the other infants (3–5 months) who were exposed to the rainy/hungry season over the second trimester of pregnancy

## Discussion

In rural Gambia, infants vaccinated during the rainy/hungry season had significantly higher antibody responses to their first DTP vaccination, compared with infants vaccinated during the dry/harvest season, despite presenting greater signs of undernutrition in early life. Of note, infants vaccinated during the rainy/hungry season spent the majority of their foetal life during the dry/harvest season months, at a point when their mother’s nutritional status was better as indicated by higher mean BMI, weight gain and mean haemoglobin levels. The timing of these effects suggests that the observed seasonal differences in infant’s antibody response to vaccination may be related to seasonal variations in maternal nutritional status during pregnancy. This is in line with the well documented critical role of prenatal nutritional exposures on the programming of the immune system and capacity to respond to vaccination in infancy ([10, 40).

Studies in both animals and humans have demonstrated that the complex immunological pathways involved in vaccine-induced antibody responses can be altered by both endogenous factors and exogenous exposures [35]. A number of factors may influence antibody responses to vaccination in infants including factors related to the vaccine itself (e.g. immunogenicity, adjuvant, dose and administration route), intrinsic host factors (sex, age, genetics and comorbidities) and extrinsic factors (diet, infections and microbiota) [39]. Moreover, maternal factors during pregnancy and lactation (e.g. nutritional status, immunisation status and infections) and perinatal factors (e.g. prematurity) may all modulate vaccine responses in infancy. The findings of our study highlight that, in rural Gambia, where many aspects of health and behaviour are dictated by a pronounced bimodal seasonality, seasonal exposures in early life may be a relevant factor in influencing antibody responses to vaccinations in infants.

Previous research highlights the importance of seasonality on vaccine responses. A systematic review published in 2015 identified 17 studies examining the impact of season of vaccination on antibody responses, including nine focusing on infant antibody responses to childhood vaccinations [26]. Seasonal variation in antibody response to the Rubella vaccine was examined in two studies: in Israel (*n* = 203) [19], the strongest Rubella antibody response was detected in infants vaccinated in the winter season, while in the Netherlands (*n* = 718), no seasonal differences were observed [8]. Four studies assessed seasonal differences in antibody responses to oral polio vaccine (OPV) in infants: in Israel (*n* = 121) [33] and India (*n* = 50) [25], the strongest antibody responses to OPV were observed in the winter season. In The Gambia (*n* = 679), however, OPV antibody responses were the highest in the dry/harvest season [20] while no seasonal variations were reported in Brazil (*n* = 730) [20]. Additionally, in The Gambia, a previous study reported seasonal trends in antibody responses to hepatitis B vaccine (HBV) in 121 infants of 52 weeks of age, and to diphtheria and tetanus vaccines included in the DTP vaccine in 138 infants at 16 weeks of age [22]. In another study from The Gambia, antibody responses to a 9-valent pneumococcal conjugate vaccine (PCV-9) were found generally higher in infants (*n* = 212) vaccinated during the rainy/hungry season [31]. Despite inconsistencies between settings and specific vaccines, the conclusions of this systematic review are in line with those of our study, indicating seasonal immunomodulation of vaccine responses in infants.

In the current study, infants vaccinated during the rainy/hungry season had significantly higher diphtheria, tetanus and pertussis antibody responses following their first DTP vaccination compared with infants vaccinated during the dry/harvest season. Tracking back to months spent in utero, all infants vaccinated during the rainy/hungry season were in mid-gestation across the dry/harvest season months. Mothers of these infants were less likely to be anaemic and underweight, had higher BMI, haemoglobin levels and gained more weight during pregnancy compared with mothers of infants vaccinated during the dry/harvest season who spent more of their pregnancy in the rainy/hungry season. These results support a possible role of seasonal variations in maternal nutritional status in shaping infant antibody responses to the DTP vaccines. Furthermore, infants with the highest antibody responses to the first DTP vaccination were the least exposed in utero to the rainy/hungry season; Their exposure was limited to a maximum duration of 2 months and split across the first and last of month gestation, which may be the least critical stages of foetal immune development.

A previously published analysis of data from the ENID trial demonstrated that maternal nutritional supplementation during pregnancy with multiple micronutrient and protein-energy improved the infant antibody response to DTP vaccinations [24]. In the current study, seasonal differences in DTP antibody responses were found to be attenuated by maternal nutritional supplementation during pregnancy. These results suggest that prenatal exposure to the rainy/hungry season during critical developmental stages may compromise immune development and the ability to mount an optimal immune response to vaccination in infancy, and that maternal nutritional supplementation during pregnancy may help to some extent reducing these detrimental effects. Together, these findings reinforce previous evidence of the pivotal role of maternal nutrition during pregnancy on the development and function of the immune system in the offspring.

Currently, the immune processes linking seasonal exposures to vaccination responses are not fully understood. A study conducted in rural Gambia showed that infants (*n* = 138) born during the rainy/hungry season, had higher levels of leukocytes and lymphocytes compared with infants born during the dry/harvest season [6]. Similarly, a study conducted in Denmark (*n* = 700) examining the influence of season of birth on 26 different immune cells subsets and 20 cytokines and chemokines, showed that at 1 month of age, infants born in winter had the highest levels of all immune cell types and mediators while infants born in summer had the lowest levels [34]. In most vaccine responses, including those elicited by the DTP vaccine, antigen-presenting cells (APC) such as B-lymphocytes, dendritic cells and macrophages are activated by vaccine antigens (e.g. diphtheria and tetanus toxoids or whole killed pertussis bacteria), initiating a cascade of processes involving many components of the immune system. Therefore, a seasonal effect on the development of immune cells involved in vaccine-induced antibody responses may provide a mechanistic explanation for the better DTP vaccine responses in infants born and vaccinated during the rainy/hungry season observed in our study.

Following three doses of the DTP vaccine, seasonal variations in vaccine responses at 24 weeks of infant age were opposite to those observed at 12 weeks; infants vaccinated during the rainy/hungry season who presented the highest diphtheria vaccine responses at 12 weeks had the lowest vaccine response at 24 weeks. Interestingly, although these infants had more favourable nutritional exposures during foetal development compared with infants vaccinated during the dry/harvest season, they were also the most exposed to the rainy/hungry season in early life and were more likely to present signs of undernutrition at 12 weeks of age, at the time of the second DTP vaccination.

A possible explanation for these results is that whilst infant nutritional status may have a limited effect on vaccine-specific antibody titres, it may have an impact on long-term immunity and recall responses. It has been shown that malnutrition may not impact the number of circulating lymphocytes and that the majority of undernourished children are capable of mounting an immune response to vaccination [27]. However, the thymus gland, which is critical for the maturation of T-lymphocytes and the formation of memory T-cells, seems affected by malnutrition even in mild cases of undernourishment, putting this organ forward as a hallmark of nutritional status [30]. Notably, in rural Gambia, a smaller thymus and lower numbers of thymus-derived T-lymphocytes were measured in infants during the rainy/hungry season compared with the dry/harvest season [5]. It has been shown in animal studies that malnutrition has a detrimental impact on the homeostatic proliferation of CD8^+^ memory T-lymphocytes and recall responses following vaccine challenges [14]. These findings suggest that the lower diphtheria vaccine responses observed in our study at 24 weeks, in infants vaccinated during the rainy/hungry season, may be explained by their exposure to the rainy/hungry season in early life. This exposure may have compromised their immune system and the induction of immune memory following vaccination challenge, resulting in an impaired recall response.

Conversely to diphtheria vaccine responses, pertussis vaccine responses at 24 weeks remained significantly higher in infants exposed to the dry/harvest season at mid-gestation and no differences in pertussis vaccine responses were observed by season of vaccination. This result may be explained by the rate of loss of antibodies taking longer to appear for pertussis vaccine responses. Antibody decay is influenced by the nature of the vaccine itself; while pertussis vaccine antigen consists of a whole killed pertussis bacteria, diphtheria and tetanus vaccines contain toxoids (inactivated toxins) secreted by the bacteria. Previous studies suggest that pertussis vaccine antigens can induce CD4^+^ and CD8^+^ T-cells responses which may result in a slower loss of antibodies and confer longer-term immunity [32].

Our results show that tetanus vaccine responses measured in infants at 24 weeks did not differ by season of gestation or vaccination. This may be related to the high immunogenicity of tetanus toxoid vaccine; after only one dose of tetanus vaccine over 95% of infants presented protective tetanus antibody levels compared to about half of infants for diphtheria and pertussis vaccines, and virtually all infants presented protective tetanus antibody titres after three doses of the vaccine. The prime-boost strategy is often implemented in vaccination programmes to increase vaccine responses and allow all subjects to reach protective antibody levels [1]. Therefore, the elevated tetanus antibody titres along with the effects of recall responses may conceal seasonal pattern in tetanus vaccine responses.

Although the results of this study suggest a strong link between seasonal variation in nutritional status and seasonal differences in DTP vaccine responses in infants, other seasonally-driven factors known to impact on immunity may be also involved. These include circulating vaccine antigens in the environment [37], sun exposure and vitamin D status [16], toxin exposure − notably aflatoxin exposure in this setting− [4] and air pollution [13], warranting further research. Further, it was interesting to note that, in spite of the known seasonality of infections in this environment [3], incidence of both maternal and infant morbidity did not vary by season (Table 1) and did not impact on the associations observed.

This study presents several strengths. Firstly, diphtheria, tetanus and pertussis antibody responses were measured in large groups of infants at 12 (*n* = 710) and 24 (*n* = 662) weeks of age. Therefore, 4 and 8 weeks after the first and third vaccinations, respectively, enabling the assessment of antibody responses within short periods following vaccinations. This minimised the effects of other potential factors which could influence vaccine responses in the intermediary period as done in some studies several years after the first initial vaccine challenge [26]. Secondly, the comparison by both seasons of mid-gestation and vaccination enabled the investigation of how seasonal exposures during pregnancy and early life may influence vaccine responses in infants. Finally, the analytical models presented were strengthened by the inclusion of carefully measured maternal and infant confounding factors.

Several limitations of this study should also be noted. This study by its observational design does not allow to draw causation but only to highlight associations between seasonally-driven factors and seasonal differences in infants’ vaccine responses. The lack of maternal vaccination status or infant pre-vaccination antibody titres are further important limitations, however, the randomisation procedure may mitigate the potential effects of these factors. Another limitation of this study is the interpretation of the effects of seasonality on immune responses measured after the three doses of the DTP vaccine, at 24 weeks of infant age, which as discussed may be concealed by the immunological effects of repeated challenges. Furthermore, at 24 weeks, infant antibody responses to DTP vaccine were measured 8 weeks following their last vaccination while they were measured 4 weeks after their first vaccination. This difference leaves a greater period for other factors such as antibody decay which may vary depending on the vaccine antigens, to mitigate and conceal the effects of seasonality on vaccine-induced antibody responses.

## Conclusion

Our findings from rural Gambia suggest that seasonal variations in primary DTP antibody responses in infants may be driven by prenatal exposure to the annual rainy/hungry season, supporting previous evidence of a critical role played by maternal nutritional status during pregnancy on the ontogeny of the immune system and the capacity of the infant to mount an optimal immune response to vaccination. With the threats of climate change, pandemics and a rapidly growing rural population, considerations of seasonality into vaccination programmes and effective nutrition-specific and agricultural-orientated interventions are urgently needed to break the vicious seasonal cycle of poverty, malnutrition and disease in rural sub-Saharan Africa.

## Supplementary Information

**Additional file 1: Figure S1.** Design of the antenatal phase of the trial. **Table S1.** Nutritional composition of the allocated daily intake of pregnancy supplements*.* **Table S2.** Mean diphtheria, tetanus and pertussis antibody titres in infants at 12 and 24 weeks of age by season of mid-gestation and first DTP vaccination*.* **Table S3.** Percentage (n (%)) of infants with protective antibody levels against diphtheria, tetanus and pertussis at 12 and 24 weeks of age by mid-gestation and vaccination seasons*.* **Figure S2.** Comparisons of mean concentrations (95% confidence intervals) of diphtheria, tetanus and pertussis antibodies by season of vaccination and maternal nutritional supplementation groups.

## Data Availability

We confirm that all data are available upon request via the research governance office at the MRC Unit The Gambia at the London School of Hygiene and Tropical Medicine (mg.crm@ccs).

## Abbreviations

APC: Antigen presenting cells
BCG: Bacille Calmette-Guerin
BMI: Body Mass Index
DSS: Demographic Surveillance System
DTP: Diphtheria-Tetanus-Pertussis
Dtxd: Diphtheria toxoid
ENID: Early Nutrition and Immune Development
FAO: Food and Agriculture Organisation
EBF: Exclusive breastfeeding
EPI: Expanded Programme of Immunisations
FeFol: Iron Folic Acid
Hb: Haemoglobin
HBV: Hepatitis B vaccine
Hib: Haemophilus influenza type B
LBW: Low birth weight
LNS: Lipid-based nutritional supplement
MMN: Multiple micronutrients
MRC: Medical Research Council
MUAC: Mid-upper arm circumference
OPV: Oral Polio Vaccine
PCV: Pneumococcal conjugate vaccine
PE: Protein energy
Ptx: Pertussis toxin
RDA: Recommeded dietary allowance
SGA: Small for gestational age
Ttx: Tetanus toxin
UNICEF: United Nations Children’s Fund
UNU: United Nations University
WHO: World Health Organisation
WLZ: Weight-for-length z-scores
