# Supplementary material for: Seasonal modulation of antibody response to diphtheria-tetanus-pertussis vaccination in infants: a cohort study in rural Gambia
Source: BMC Public Health. 2021 Jul 22;21:1442. doi: 10.1186/s12889-021-11383-7 (PMC8296693; doi:10.1186/s12889-021-11383-7)
Supplement: Supplementary file 1 — Additional file 1: Figure S1. Design of the antenatal phase of the trial. Table S1. Nutritional composition of the allocated daily intake of pregnancy supplements. Table S2. Mean diphtheria, tetanus and pertussis antibody titres in infants at 12 and 24 weeks of age by season of mid-gestation and first DTP vaccination. Table S3. Percentage (n (%)) of infants with protective antibody levels against diphtheria, tetanus and pertussis at 12 and 24 weeks of age by mid-gestation and vaccination seasons. Figure S2. Comparisons of mean concentrations (95% confidence intervals) of diphtheria, tetanus and pertussis antibodies by season of vaccination and maternal nutritional supplementation groups. [file 12889_2021_11383_MOESM1_ESM.docx]

**
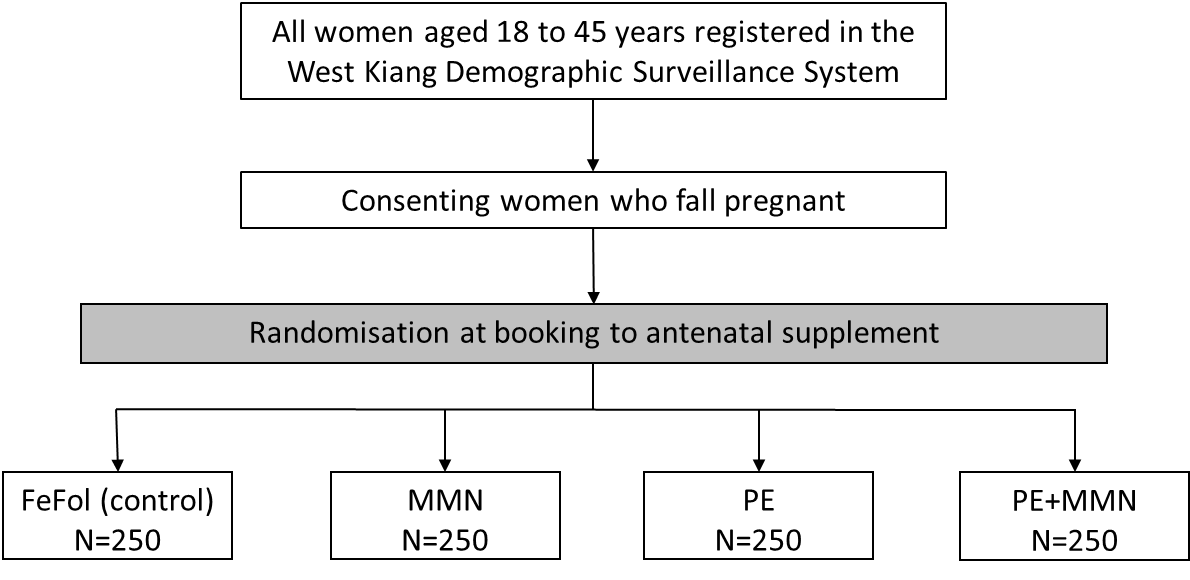
**

**Figure S1. Design of the antenatal phase of the trial.** FeFol, iron folic acid; MMN, multiple micronutrients and PE, protein energy.

**Table S1. Nutritional composition of the allocated daily intake of pregnancy supplements**

|  | **Tablets** | | **LNS** | |
| --- | --- | --- | --- | --- |
| **Nutrients** | **FeFol** | **MMN** | **PE** | **PE + MMN** |
| Iron (mg) | 60 | 60 | 60 | 60 |
| Folate (μg) | 400 | 400 | 400 | 400 |
| Vitamin A (RE μg) |  | 1600 | *2.85* | 1600 |
| Vitamin D (IU) |  | 400 | *-* | 400 |
| Vitamin E (mg) |  | 20 | *4.2* | 20 |
| Vitamin C (mg) |  | 140 | *2.25* | 140 |
| Vitamin B1 (mg) |  | 2.8 | *0.3* | 2.8 |
| Vitamin B2 (mg) |  | 2.8 | *0.45* | 2.8 |
| Niacin (mg) |  | 36 | *1.35* | 36 |
| Vitamin B6 (mg) |  | 2.8 | *0.15* | 2.8 |
| Vitamin B12 (μg) |  | 5.2 | *0.1* | 5.2 |
| Zinc (mg) |  | 30 | *3.3* | 30 |
| Copper (mg) |  | 4 | *1.05* | 4 |
| Selenium (μg) |  | 130 | *6.15* | 130 |
| Iodine (μg) |  | 300 | *2.6* | 300 |
| Energy (kcal) |  |  | 746 | 746 |
| Protein (g) |  |  | 20.8 | 20.8 |
| Lipids (g) |  |  | 52.6 | 52.6 |

FeFol, iron folic acid; MMN, multiple micronutrients; PE, protein energy; LNS, lipid-based nutrient supplement.

Data in italics represent natural micronutrients content in PE which is made from the food base ingredients.

**Table S2. Mean diphtheria, tetanus and pertussis antibody titres in infants at 12 and 24 weeks of age by season of mid-gestation and first DTP vaccination**

|  | **Season of mid-gestation** | | | |  | **Season of vaccination** | | | |
| --- | --- | --- | --- | --- | --- | --- | --- | --- | --- |
|  | **Unadjusted models** | | | |  | **Unadjusted models** | | | |
| **Vaccine** | **Dry/Harvest**  **(n = 335)** | **Rainy/Hungry**  **(n = 375)** | **Effect size (95%CI)^a^** | ***p-value^b^*** |  | **Dry/Harvest**  **(n = 486)** | **Rainy/Hungry**  **(n = 224)** | **Effect size (95%CI)^a^** | ***p-value^b^*** |
| **12 weeks** |  |  |  |  |  |  |  |  |  |
| Diphtheria | 0.19 (0.15, 0.24) | 0.08 (0.07, 0.10) | 35.6 (23.7, 47.5) | **<0.001** |  | 0.09 (0.08, 0.10) | 0.26 (0.20, 0.35) | -48.1 (-60.7, -35.4) | **<0.001** |
| Tetanus | 0.74 (0.67, 0.82) | 0.55 (0.50, 0.61) | 12.6 (6.4, 18.7) | **<0.001** |  | 0.58 (0.53, 0.63) | 0.78 (0.69, 0.88) | -13.2 (-19.8, -6.6) | **<0.001** |
| Pertussis | 6.3 (5.5, 7.1) | 4.9 (4.3, 5.6) | 10.9 (3.2, 18.7) | **0.006** |  | 4.9 (4.4, 5.5) | 7.1 (6.1, 8.2) | -16.3 (-24.5, -8.0) | **<0.001** |
| **24 weeks** |  |  |  |  |  |  |  |  |  |
| Diphtheria | 1.2 (1, 1.3) | 1.6 (1.5, 1.8) | -14.7 (-21.2, -8.1) | **<0.001** |  | 1.6 (1.5, 1.7) | 1.0 (0.86, 1.2) | 19.6 (12.6, 26.7) | **<0.001** |
| Tetanus | 3.7 (3.2, 4.3) | 3.8 (3.4, 4.2) | -1.0 (-8.8, 6.7) | 0.791 |  | 3.8 (3.5, 4.2) | 3.9 (3.2, 4.7) | -1.1 (-9.3, 7.2) | 0.800 |
| Pertussis | 106.2 (86.2, 130.7) | 82.8 (67.5, 101.5) | 10.8 (-1.9, 23.5) | 0.094 |  | 91.6 (76.6, 109.7) | 92.6 (71.7, 119.4) | -0.40 (-14.1, 13.3) | 0.950 |

^a^Effect sizes were determined using the mean difference between the dry season and rainy season of vaccination from the Student’s *t-*test and were expressed as percentages (%).

^b^The p-values were calculated by Student’s *t*-test on the log-transformed antibody concentrations.

**Table S3. Percentage (n (%)) of infants with protective antibody levels against diphtheria, tetanus and pertussis at 12 and 24 weeks of age by mid-gestation and vaccination seasons**

|  | **Mid-gestation season** | | | **Vaccination season** | | |
| --- | --- | --- | --- | --- | --- | --- |
| **Vaccine^a^** | **Dry/Harvest**  **(Nov-May) (n = 335)** | **Rainy/Hungry**  **(Jun-Oct) (n = 375)** | ***p-value^b^*** | **Dry/Harvest**  **(Nov-May) (n = 486)** | **Rainy/Hungry**  **(Jun-Oct) (n = 224)** | ***p-value^b^*** |
| **12 weeks** |  |  |  |  |  |  |
| Diphtheria | 211 (63.0) | 183 (48.8) | **<0.001** | 244 (50.2) | 150 (67.0) | **<0.001** |
| Tetanus | 3331 (98.8) | 360 (96.0) | **0.021** | 470 (96.7) | 221 (98.7) | 0.134 |
| Pertussis | 207 (61.8) | 199 (53.1) | **0.019** | 253 (52.1) | 153 (68.3) | **<0.001** |
| **24 weeks** |  |  |  |  |  |  |
| Diphtheria | 285 (93.8) | 337 (99.7) | **<0.001** | 435 (99.5) | 187 (91.2) | **<0.001** |
| Tetanus | 302 (99.3) | 337 (99.7) | 0.502 | 436 (99.8) | 203 (99.0) | 0.196 |
| Pertussis | 277 (91.1) | 298 (88.2) | 0.222 | 390 (89.2) | 185 (90.2) | 0.699 |

^a^Presenting protective antibody levels and therefore being respondent to the vaccine was defined as having an antibody titre >0.1 IU/mL for diphtheria and tetanus, according to international standards (WHO). An arbitrary threshold was established at >5.0 EU/mL as for pertussis an in-house antibody assay was used.

^b^The p-values were calculated by Student’s *t*-test.


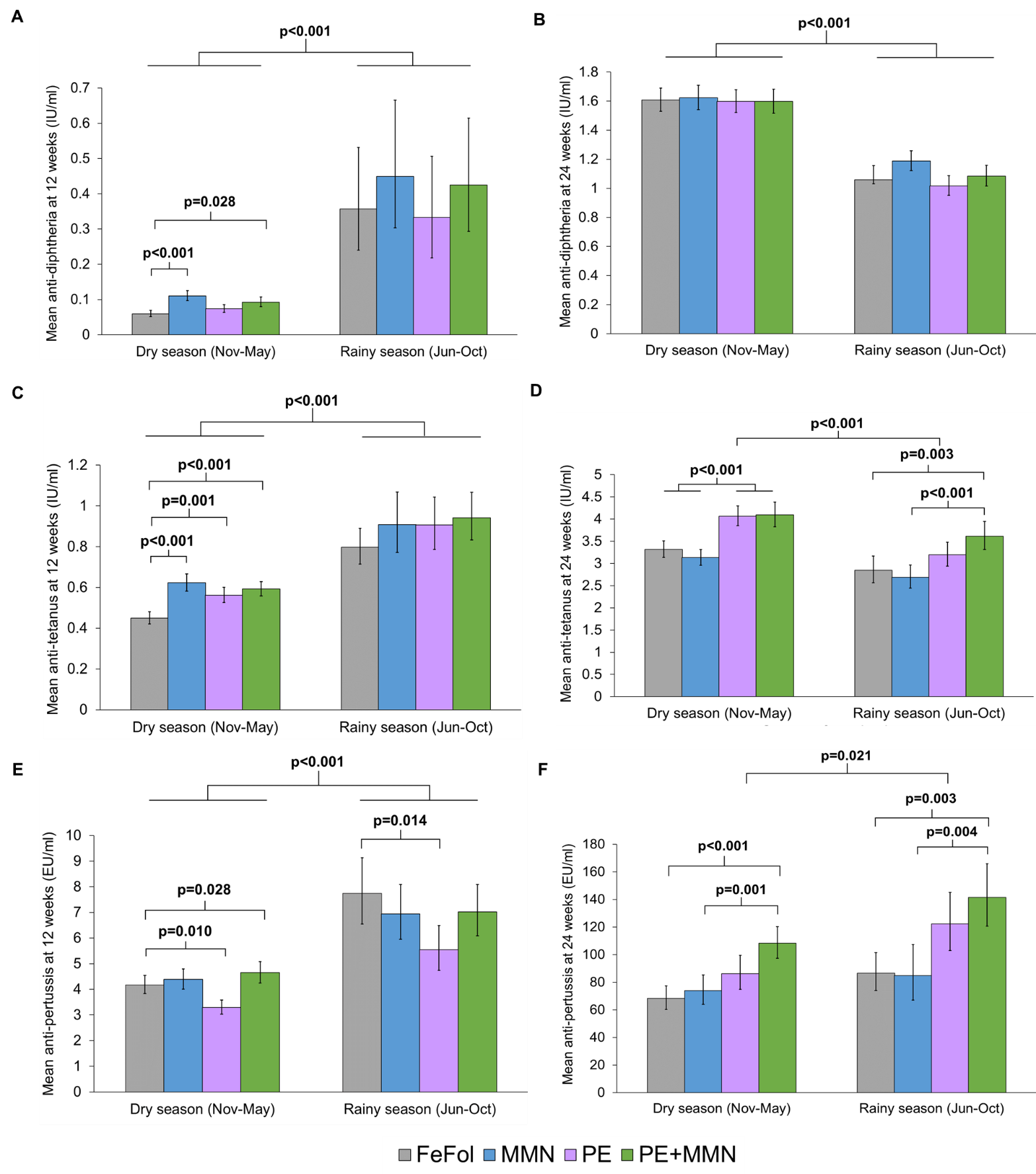


**Figure S2. Comparisons of mean concentrations (95% confidence intervals) of diphtheria, tetanus and pertussis antibodies by season of vaccination and maternal nutritional supplementation groups.** Antibody titres were measured at 12 weeks after a single dose of DTP vaccines and at 24 weeks after three doses of the vaccines. Means were adjusted with maternal variables; supplement group, compliance to supplement, age, haemoglobin levels, formal education, morbidity, and BMI, and with infant variables; gestational age at delivery, sex, WLZ at 8 weeks of age, haemoglobin levels at 12 weeks, morbidity, exclusively breastfed, birth season and Fourier terms of month of mid-gestation or first vaccination. For the 24 weeks data, means were adjusted with the same factors mentioned above with changes for: infant WLZ at 16 weeks, haemoglobin levels at 24 weeks, morbidity and exclusively breastfed from birth to 24 weeks. Means were back-transformed from the log-scale and expressed in IU/ml for diphtheria and tetanus antibody titres and in EU/ml for pertussis antibody titres. P-values were calculated by t-test for each comparison.

Abbreviations: Iron folic-acid (reference); MMN, multiple micronutrient; PE, protein-energy, PE+MMN, protein energy combined with multiple micronutrients.
